# Artificial intelligence-assisted diagnosis of ocular caruncle oncocytoma: a proof- of-concept case report of two cases

**DOI:** 10.3389/fopht.2026.1772378

**Published:** 2026-04-24

**Authors:** Matteo Sacchi, Clara Ellecosta, Sara Sechi, Stefano Dore, Antonio Pinna

**Affiliations:** 1 Department of Medicine, Surgery and Pharmacy, University of Sassari, Sassari, Italy; 2 Eye Clinic, Azienda Ospedaliera Universitaria - University of Sassari, Sassari, Italy

**Keywords:** caruncle tumor, case report, ChatGPT, images analysis, large language model, oncocytomas, slit-lamp photographs

## Abstract

**Background:**

Oncocytomas of the ocular caruncle are rare benign epithelial tumors. Their clinical diagnosis is challenging, as they can mimic other benign or malignant lesions such as papilloma, nevus, squamous cell carcinoma, melanoma, or oncocytic carcinoma. For this reason, histopathological confirmation remains indispensable. The aim of this study was to test the ability of a multimodal large language model (ChatGPT, GPT-5, 2025 version) to generate diagnostic hypotheses directly from slit-lamp images, supported by brief clinical summaries.

**Case presentation:**

We retrospectively analyzed two cases of caruncular oncocytoma that had undergone surgical excision with subsequent histopathological confirmation. For each case, ChatGPT was provided only with slit-lamp photographs of the lesion and a concise clinical summary including age, sex, and the site of the lesion (caruncle). No histopathological data or additional clinical details were supplied. In both cases, ChatGPT proposed oncocytoma as the primary diagnostic hypothesis. The model also generated differential diagnoses including papilloma, nevus, as well as the possibility of a malignant lesion such as squamous cell carcinoma or melanoma.

**Conclusions:**

This proof-of-concept demonstrates, for the first time to our knowledge, that a general- purpose multimodal AI system can correctly recognize a rare ocular surface tumor from slit-lamp images. While preliminary and limited by the very small sample size, these findings suggest that large language models may assist clinicians in considering rare adnexal tumors during differential diagnosis. Further research on larger datasets is required, and histopathology will remain the gold standard for definitive diagnosis.

## Background

Oncocytomas are rare benign epithelial neoplasms composed of oncocytic cells with abundant eosinophilic granular cytoplasm due to mitochondrial hyperplasia. They most frequently occur in kidneys, salivary glands, and thyroid, while ocular adnexal oncocytomas are exceptional, with the caruncle being the most commonly affected site (1, 5, 6). Although the caruncle is the most frequent site, oncocytic tumors have rarely been reported in other ocular adnexal structures, including the conjunctiva and lacrimal drainage system (5). In large clinicopathologic series, oncocytomas account for only a small minority of caruncular lesions (approximately 4%) and typically present as small, slow-growing nodules with a mean base of 4.1 mm (6, 7). The clinical diagnosis of ocular oncocytoma is difficult, as its appearance can closely resemble a variety of benign and malignant lesions, including papillomas, nevi, squamous cell carcinoma, melanoma, and oncocytic carcinoma (1, 4, 8). For this reason, histopathological confirmation remains the gold standard for diagnosis. Artificial intelligence (AI), particularly deep learning, has gained increasing importance in ophthalmology, especially in retinal disease detection (2, 3). More recently, its role has expanded into oculoplastics as well: a major systematic review evaluated 91 published studies, demonstrating that AI systems have been applied to ptosis assessment, periocular and orbital tumors, orbital fractures, inflammatory and autoimmune orbital diseases, and thyroid eye disease, reporting consistently strong diagnostic performance across multiple domains (9). Recent reviews focusing on ocular adnexal tumors have emphasized the importance of structured validation datasets and comparator cohorts for evaluating AI-based diagnostic tools (10). In parallel, large language models (LLMs) are emerging as diagnostic support tools across medical specialties. ChatGPT is a multimodal LLM developed by OpenAI, capable of processing both images and clinical text. However, unlike task-specific imaging algorithms trained on curated datasets and evaluated with quantitative performance metrics, multimodal LLMs are general-purpose systems that generate probabilistic differential diagnoses and are not specifically calibrated for adnexal tumor classification. In this proof-of-concept report, we describe two cases of histologically confirmed ocular oncocytoma and evaluate the ability of ChatGPT (GPT-5, 2025) to generate diagnostic hypotheses based solely on slit-lamp images and minimal clinical information.

## Case presentation

This study was designed as a retrospective proof-of-concept analysis.

Two patients with histopathologically confirmed oncocytoma of the ocular caruncle were included. Both cases had undergone complete ophthalmological examination, surgical excision of the lesion, and histopathological confirmation of the diagnosis.

This was an exploratory diagnostic support evaluation conducted on de-identified clinical images and case descriptions. The study adhered to the principles of the Declaration of Helsinki. Since the analysis was retrospective, non-interventional, and performed on anonymized data, formal ethics board approval was not required.

After histopathological confirmation, the two cases were retrospectively re-evaluated using ChatGPT, a large multimodal language model (GPT-5, OpenAI, 2025 version), capable of processing both natural language and clinical images. For each case, the model was provided with slit-lamp photographs of the caruncular lesion and a brief clinical summary including patient age, sex, and lesion site. The images were acquired during routine ophthalmic examination using a TAKAGI slit-lamp connected to a computer equipped with the EyeGest imaging software, which allows digital acquisition and storage of slit-lamp photographs. Images were obtained at 10× magnification and saved in JPEG format at their original resolution. No post-acquisition editing or image enhancement was performed prior to AI analysis. A standardized prompt was used for both cases:

“Medical project, ophthalmology. Male patient, approximately 75 years old, with a caruncular lesion. Could you analyze the image and provide a diagnostic hypothesis?”.

The model was queried once per case using a standardized prompt. Repeated runs were not performed, as the objective of this study was exploratory feasibility rather than assessment of output stability.

No information regarding the definitive histopathological diagnosis was given to the model. The order of the diagnostic hypotheses was recorded, with particular attention to whether oncocytoma was included and its position among the proposed diagnoses.

To further explore the robustness of the model’s diagnostic reasoning, we conducted two additional exploratory analyses. First, we performed an exploratory analysis using images of three additional caruncular tumors reported in the literature, including basal cell carcinoma, marginal zone lymphoma, and squamous cell carcinoma. The same prompt and analytical procedure used for the oncocytoma cases were applied. Second, in a separate exploratory step, the slit-lamp images of our two oncocytoma cases and clinical prompt were analyzed using another publicly available artificial intelligence platform (Perplexity AI). This analysis was not intended as a formal comparison between AI systems but was performed to explore whether a similar differential diagnostic reasoning could be generated by another AI platform.

The study was not designed to compare AI-generated hypotheses with independent clinician assessments or to calculate quantitative diagnostic performance metrics. Rather, the objective was to explore the feasibility of generating clinically plausible differential diagnoses in the context of a rare ocular tumor.

## Patient information

### Case 1

An 84-year-old male presented with a slowly growing, asymptomatic lesion of the right ocular caruncle, first noticed approximately four years before presentation.

### Case 2

A 69-year-old male presented with a lesion of the right ocular caruncle, first noticed approximately four months before presentation. His ocular history included chalazion surgery in the same eye approximately thirty years earlier.

A chronological timeline summarizing the main clinical events for both cases is provided in **Table 1**.

**Table 1:** Timeline of the two cases.

Event	Case 1	Case 2
Onset lesion	4 years before presentation	4 months before presentation
First ophthalmic evaluation	29/11/2024	10/03/2025
Slit-lamp imaging	29/11/2024	10/03/2025
Surgical excision	27/01/2025	26/03/2025
Histopathological diagnosis	11/02/2025	04/04/2025
AI analysis	08/09/2025	08/09/2025

## Clinical findings

### Case 1

Ophthalmological examination revealed a reddish, well-circumscribed nodular lesion measuring approximately 0.5 cm, located in the right ocular caruncle.

### Case 2

Slit-lamp examination showed a vascularized lesion of the right ocular caruncle.

## Diagnostic assessment

Both lesions were surgically excised and submitted for histopathological evaluation.

In Case 1, histopathological examination revealed stratified squamous epithelium overlying solid nests and cystic structures composed of oncocytic epithelial cells, consistent with a benign oncocytoma.

In Case 2, histopathology demonstrated a non-encapsulated proliferation of oncocytic epithelial cells arranged in cystic, solid, and papillary patterns, confirming the diagnosis of benign oncocytoma.

The diagnostic hypotheses generated by the model for both cases are analyzed descriptively, with particular attention to whether oncocytoma was included and its rank among the proposed differential diagnoses as summarized in **Table 2**.

**Table 2:** AI diagnostic hypotheses.

Case 1	Case 2
*oncocytoma*	*oncocytoma*
papilloma	epithelial cyst/pseudocyst
pseudocyst/dermoid/lipodermoid	Papilloma
malignant lesion (squamous cell carcinoma)	malignant lesion (squamous cell carcinoma, melanoma, linfoma)

**Figure 1:**
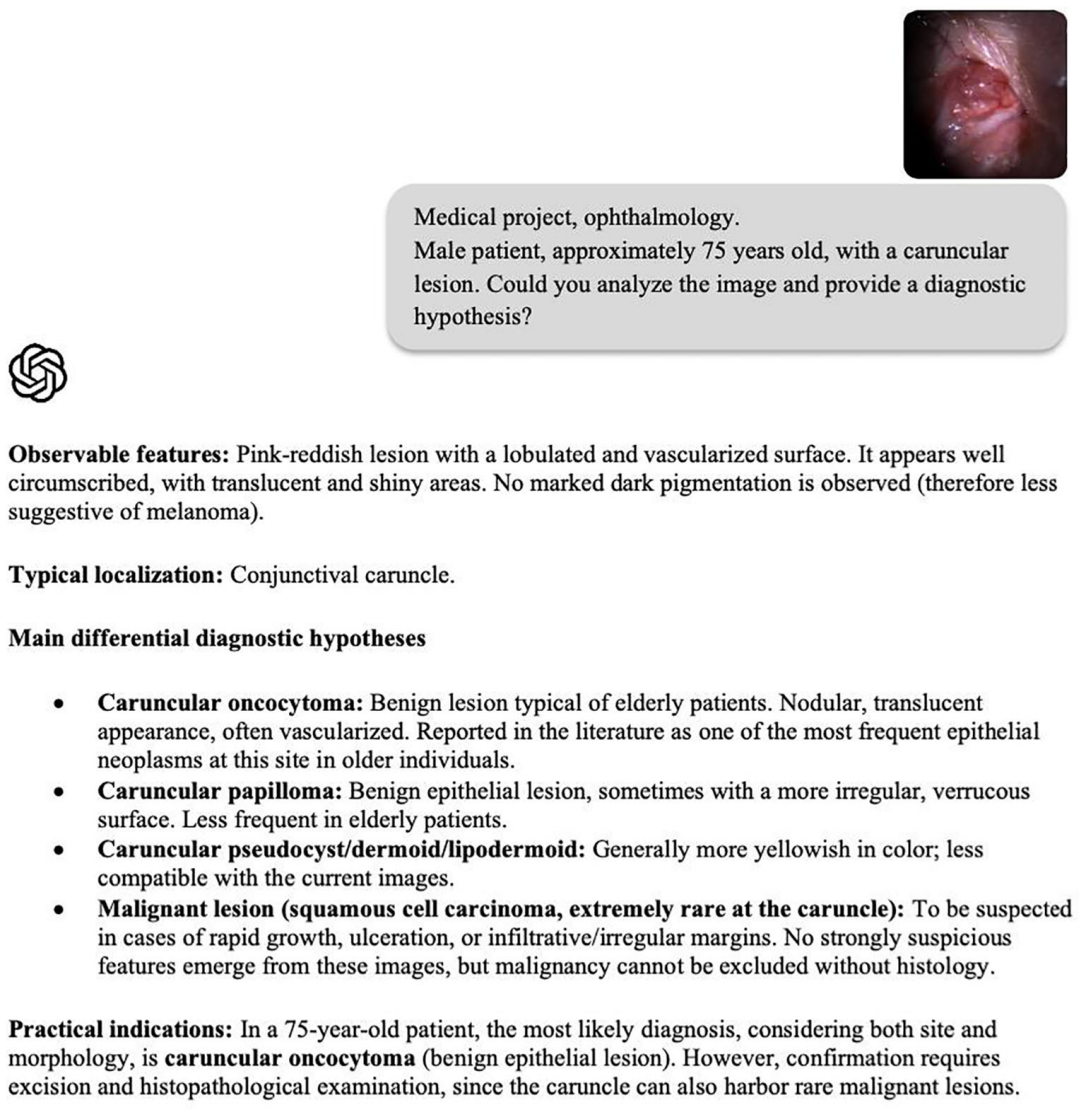
Slit-lamp photograph of a vascularized caruncular lesion (Case 1). ChatGPT (GPT-5, 2025 multimodal version), provided with only this image and a short clinical summary, suggested oncocytoma as the primary diagnostic hypothesis, followed by papilloma and the possibility of a malignant lesion (squamous cell carcinoma or melanoma).

In Case 1, the model proposed oncocytoma as the primary diagnostic hypothesis, followed by papilloma, other benign lesions, and squamous cell carcinoma (**Figure 1**; **Table 2**). In Case 2, ChatGPT suggested oncocytoma as the first hypothesis, followed by epithelial cyst/pseudocyst, papilloma, and malignant lesions including squamous cell carcinoma, melanoma, and lymphoma.

In both cases, the model explicitly stated that confirmation requires surgical excision and histopathological examination, emphasizing the need for histological confirmation given the potential for malignant lesions of the caruncle.

The exploratory analyses yielded the following findings. When the same prompt was applied to images of other caruncular tumors, the model proposed diagnostic hypotheses different from the confirmed diagnosis. In particular, apocrine hidrocystoma was suggested for basal cell carcinoma (12), oncocytoma appeared as the main diagnostic hypothesis for marginal zone lymphoma (13), and squamous papilloma was proposed as the most likely diagnosis for squamous cell carcinoma (14). Oncocytoma was also included among the differential diagnoses for the squamous cell carcinoma (**Table 3**).

**Table 3:** Exploratory analysis of additional caruncular tumors from the literature tested with ChatGPT using the same prompt.

Lesion	Source	ChatGPT main hypothesis	Other suggested diagnoses
Basal cell carcinoma	Ref 12	Apocrine hidrocystoma	Epithelial inclusion cyst, oncocytoma
Marginal zone lymphoma	Ref 13	Oncocytoma	Conjunctival epithelial inclusion cyst, Pyogenic granuloma
Squamous cell carcinoma	Ref 14	Squamous papilloma	Oncocytoma, conjunctival nevus, amelanotic melanoma

In the second exploratory analysis, the slit-lamp images of our two oncocytoma cases and the identical prompt were analyzed using Perplexity AI. In this case, the system suggested pyogenic granuloma as the most likely diagnosis, while oncocytoma was included among the differential diagnoses together with other benign and malignant entities (**Table 4**). These findings indicate that different AI systems may generate partially overlapping but not identical diagnostic hypotheses.

**Table 4:** Comparison of diagnostic hypotheses generated by ChatGPT and Perplexity AI.

AI system	Primary diagnosis	Differential diagnoses
ChatGPT	Oncocytoma	papilloma, SCC, nevus
Perplexity AI	Pyogenic granuloma	oncocytoma, papilloma, malignant lesion

## Therapeutic intervention

In both cases, complete surgical excision of the caruncular lesion was performed under local anesthesia.

## Follow-up and outcomes

Postoperative recovery was uneventful in both patients. During follow-up, no clinical evidence of local recurrence or complications was observed.

## Patient perspective

A patient-reported perspective was not available for inclusion in this report.

## Conclusions

In this work, we tested the ability of ChatGPT (GPT-5, 2025) to recognize two histologically confirmed cases of ocular caruncle oncocytoma based on slit-lamp photographs and brief clinical summaries. To our knowledge, this is the first proof-of- concept demonstration that a multimodal large language model can correctly identify a rare ocular tumor such as caruncular oncocytoma from slit-lamp images and a brief clinical summary. Ocular oncocytomas are exceedingly rare, with the majority of reported cases involving the caruncle. While most case series describe them in elderly female patients, with a female-to-male ratio of approximately 3:1 and a peak incidence in the seventh decade of life (5, 7), our two cases involved male patients aged 69 and 84, both with prior ocular interventions and a history of smoking—possible predisposing factors worth exploring further. In large clinicopathologic reviews, nevi and papillomas represent the most frequent caruncular lesions, while oncocytomas account for only a small minority (≈4%) (6). They typically affect elderly patients, with a female predominance, and are usually small, slow-growing, and asymptomatic (5, 7). However, larger or cystic variants have also been reported (7). Histologically, oncocytomas are composed of oncocytic epithelial cells arranged in glandular or solid patterns and show strong immunoreactivity for cytokeratins, epithelial membrane antigen, and mitochondrial markers (5).

The most notable finding of our study is that ChatGPT (GPT-5 multimodal, 2025), when provided with slit-lamp photograph and minimal clinical history, proposed oncocytoma as the first diagnostic hypothesis in both cases. This result is remarkable given the rarity of the tumor and the absence of explicit training data on such lesions. In an exploratory step, the same images and clinical prompt were also analyzed using Perplexity AI. While this system suggested pyogenic granuloma as the primary hypothesis, oncocytoma was still included among the differential diagnoses. This observation further illustrates that different AI systems may generate partially overlapping but non-identical diagnostic hierarchies, highlighting the probabilistic nature of AI-generated diagnostic reasoning.

From a clinical perspective, the importance of this result lies in the differential diagnosis. The caruncle can host a variety of benign and malignant lesions, including papilloma, nevus, squamous cell carcinoma, oncocytic carcinoma, and melanoma (4, 6, 8). Among these, conjunctival melanoma is particularly critical, given its malignant potential and poor prognosis in caruncular involvement. Importantly, pigmented oncocytomas may clinically resemble melanoma, sometimes leading to referral with a presumptive diagnosis of malignancy (4). This underscores the indispensable role of histopathology for definitive diagnosis.

These proof-of-concept findings suggest potential roles for AI in ophthalmology:

1. Decision support: assisting clinicians in expanding the differential diagnosis, especially in rare or ambiguous cases.
2. Educational aid: offering suggestions that may sensitize trainees and general ophthalmologists to uncommon entities like oncocytoma.
3. Safety net for malignancies: highlighting differential diagnoses such as melanoma, ensuring they are considered early in the diagnostic pathway.

This study has limitations that must be acknowledged. First, the sample size was extremely limited (N = 2), and the results cannot be generalized to routine clinical practice. The aim of this work was strictly proof-of-concept, intended to explore feasibility rather than to provide definitive diagnostic evidence. Although an exploratory analysis using a small number of additional caruncular tumors retrieved from the literature was performed, this limited assessment does not allow a systematic evaluation of susceptibility to confounders. In this exploratory test, the model generated variable diagnostic hypotheses: oncocytoma appeared as the primary diagnostic hypothesis for marginal zone lymphoma and as a possible alternative diagnosis for squamous cell carcinoma. These observations suggest that, under conditions of minimal clinical input, the model may occasionally rank oncocytoma among the differential diagnoses of lesions with overlapping clinical features. Consequently, potential false-positive oncocytoma suggestions cannot be excluded. However, given the very limited number of tested images, the present study was not designed to evaluate specificity, diagnostic stability, or consistency across different lesion types. Second, the AI system was intentionally kept blind, being provided only with slit-lamp photographs and a brief clinical summary, without access to histological data or further clinical information. Third, large language models such as ChatGPT may generate so-called “hallucinations”, producing plausible yet factually incorrect outputs. In addition, their responses lack verifiable source attribution, which limits transparency and traceability. These intrinsic limitations of the technology must be considered when interpreting the results of this study. Furthermore, large language models generate probabilistic outputs that may vary depending on prompt formulation, image quality, and contextual input. As such, reproducibility and output stability cannot be inferred from two isolated cases. Structured validation studies including repeated runs and standardized prompts would be required to assess consistency.

Nevertheless, important limitations remain: ChatGPT is a general-purpose model, its outputs are probabilistic and context-dependent, and histopathology remains indispensable for definitive diagnosis. Dedicated artificial intelligence models have been developed for specific ocular surface tumors, such as ocular surface squamous neoplasia, showing promising diagnostic performance under structured validation conditions (10). In contrast, large language models are general-purpose systems and are not specifically trained for adnexal tumor classification. Future studies should include larger validation datasets, standardized panels of clinically similar lesions, and benchmarking against both human experts and dedicated ophthalmic imaging AI systems, as emphasized by recent reviews in this field (11).

In conclusion, we describe two histologically confirmed cases of ocular caruncle oncocytoma in which ChatGPT (GPT-5 multimodal, 2025) correctly suggested oncocytoma as the first diagnostic hypothesis based solely on clinical images and short summaries. While histopathological confirmation remains mandatory, these findings suggest a potential role for multimodal LLMs in supporting ophthalmologists with the differential diagnosis of rare adnexal tumors. We hope this exploratory, proof-of-concept work will encourage further research on this topic.

## Data Availability

The original contributions presented in the study are included in the article/supplementary material. Further inquiries can be directed to the corresponding author.
